# Single-Track Magnetic Tape Absolute Position Sensor with Self-Adaptivity

**DOI:** 10.3390/s24134220

**Published:** 2024-06-28

**Authors:** Zoltán Kántor, Attila Szabó

**Affiliations:** Balluff-Elektronika Kft., Pápai út 55, 8200 Veszprém, Hungary; attila.szabo@balluff.hu

**Keywords:** position sensor, magnetic code tape, linear magnetic encoder, self-adaptive

## Abstract

In this study, we demonstrate a single-track magnetic code tape-based absolute position sensor system. Unlike traditional dual-track systems, our method simplifies manufacturing and avoids crosstalk between tracks, offering higher tolerance to alignment errors. The sensing system employs an array of magnetic field sensing elements that recognize the bit sequence encoded on the tape. This approach allows for accurate position determination even when the number of sensing elements is fewer than the number of bits covered, and without the need for specific spacing between sensing elements and bit length. We demonstrate the system’s ability to learn and adapt to various magnetic code patterns, including those that are irregular or have been altered. Our method can identify and localize the sensed magnetic field pattern directly within a self-learned magnetic field map, providing robust performance in diverse conditions. This self-adaptive capability enhances operational safety and reliability, as the system can continue functioning even when the magnetic tape is misaligned or has undergone changes.

## 1. Introduction

In automation applications, incremental magnetic position sensors are utilized to monitor the movement of machine parts as a sensing head traverses a periodically magnetized code tape. This sensing head typically incorporates a minimum of two single-axis, or preferably dual-axis, magnetic field sensing elements. These sensors employ quadrature decoding and resolver circuits to ensure high resolution. To establish the absolute position of the machine part, a reference run or teach-in function is typically performed upon each power-on cycle. Nevertheless, these procedures may be bypassed if the sensor head is powered by a backup source, allowing it to continue counting magnetic periods even during downtime [1]. The Wiegand effect was also shown to generate sufficient power for the operation of up and down counters without any external energy source, which gives a further opportunity to retain the absolute position information when the sensor is not powered [2,3].

Alternatively, absolute measurement solutions utilize tapes that are non-periodically magnetized [1,4,5]. These tapes may feature multiple tracks; one periodically magnetized for precise incremental measurement and another with a non-repeating code for rough localization, such as an n-bit maximum length sequence or another pseudorandom sequence. Once the coarse position is determined, the exact sensor position is established using the resolved incremental position for refinement. In [5], the proposal included multiple transversally arranged magnetic domains per unit length. In [6], two sets of binary magnetic field sensing elements, shifted relative to each other, are provided to sample the absolute code bitwise (with exactly one sensing element per magnetic bit length). The selection between the two sets and the timing of reading the selected set are determined and triggered by transitions of the sensing element responsible for reading the incremental track. Another approach to achieving absolute sensing within a limited range involves using two parallel incremental tracks with different periods, applying the nonius principle [7,8].

Approaches employing dual tracks on the magnetic code tape may encounter challenges like crosstalk and susceptibility to misalignment, imposing limitations in various applications. Ensuring operational safety requires proactive detection of escalating misalignment, preventing situations where position measurement becomes unfeasible.

There are absolute positioning systems utilizing single-track magnetic code tapes, focusing solely on the absolute track containing the nonrepeating code [9,10]. Birrer et al. [10] employed a tape where each bit was encoded by two oppositely polarized magnetic domains, ensuring homogeneous domains not exceeding one bit length. They employed an array of analog magnetic field sensing elements and a corresponding analog signal processing circuit to decode the sensed field pattern into the binary sequence covered by the sensing array. Additionally, a smaller set of sensing elements aligned with the analog decoder array generated a higher resolution signal for interpolation within one bit length. This system utilizes multiple sensing elements per bit for absolute code generation, necessitating precise spacing between sensing elements to match the bit length. Similar constraints were observed in the approach by Muller [9]. An approach to using a single-track incremental code tape for absolute position sensing was presented by Park et al. [11]. Their configuration resembles incremental sensing but incorporates both alternating magnetic polarity and a monotonous change in the magnetic field magnitude within the sensing range. This is achieved by arranging a regular incremental code tape section at a narrow angle to the sensor trajectory, producing a linearly variable distance, or by varying the width, thickness, or magnetic polarization strength of the magnetic domains. However, their position sensing system provides only a limited absolute sensing range and is highly sensitive to any misalignment.

This paper presents a position determination system featuring a magnetic code tape with a single track carrying an absolute code pattern. This approach offers easier manufacturability compared to dual-track tapes, eliminates crosstalk between tracks, and increases tolerance to alignment errors. Additionally, it demonstrates how the sensor can deduce the bit pattern covered by the sensing head, even with fewer sensing elements than covered bits and without specific spacing relative to magnetic bit width. Apart from reducing manufacturing costs associated with single-track tapes, this leads to cost savings in the sensor head and improves tolerance to positioning and alignment errors, enhancing operational safety. Furthermore, the paper illustrates how a sensor can adapt to unknown code generation algorithms or changes in the magnetic tape since its last visit. Finally, it introduces a method that imposes fewer constraints on tape construction, utilizing the sensed magnetic field pattern directly for localization within a self-learned magnetic field map.

## 2. Identification and Positioning of Magnetic Bit Patterns

**Figure 1:**
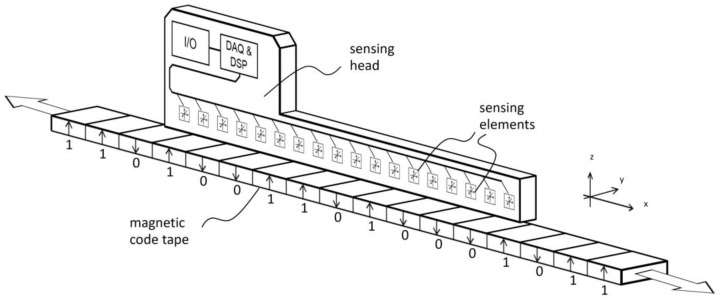
Schematic arrangement of the sensing head and the magnetic code tape. In our study, we employed 32 three-axis sensing chips in WLCSP packaging, arranged on an equidistant grid with 1.805 mm spacing. By selectively reading the chip data, we were also able to simulate reduced resolutions with chip distances of 3.61 mm, 5.415 mm, and so on.

The sensor head is made up of a collection of sensing elements; specifically, two- or three-axis magnetic field sensing chips arrayed along the sensitive *x* axis. As illustrated in Figure 1, the sensing head is positioned “over” the magnetized encoding strip where the arrows represent the magnetic polarization direction within each equally long domain. The sensor has the ability to move in relation to the encoding strip along the *x* axis. In practical terms, the measurement of the x position is essentially determining the sensor head’s position; that is, a reference point within the sensor head in the encoding strip’s coordinate system. This reference point could be the first sensing element’s position within the array or another specific point in the housing. It is useful to have the encoding strip and the sensor’s coordinate systems aligned in the same way. If the sensing array is precisely positioned in the *x*-*z* plane of the encoding strip’s coordinate system, the y components of the measured magnetic field vectors automatically become zero due to symmetry. However, if the sensor head is slightly moved sideways in the y direction or if the sensor head is rotated around its *x* axis, the y component can still be removed or minimized by rotating the measured vectors around *x* by the same angle. This reduces the problem to the *x*-*z* plane. The aforementioned rotation can be carried out autonomously when non-zero *y* components are detected autonomously, which could be a part of the sensor system’s self-installation or self-adaptation. Following this, we will address the problem in the *x*-*z* plane.

**Figure 2:**
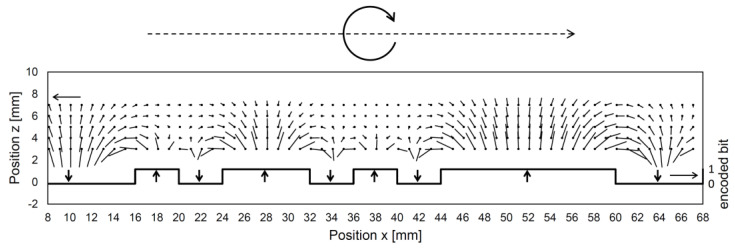
The direction and magnitude of the magnetic field vector in the space above a magnetic code tape are shown. The dots represent the points where the vectors are calculated. To simplify the illustration, the ends of the vector arrows have been omitted. The encoded bits and the corresponding magnetic polarization direction are visualized at the bottom of the plot. This figure contains simulated data.

**Figure 3:**
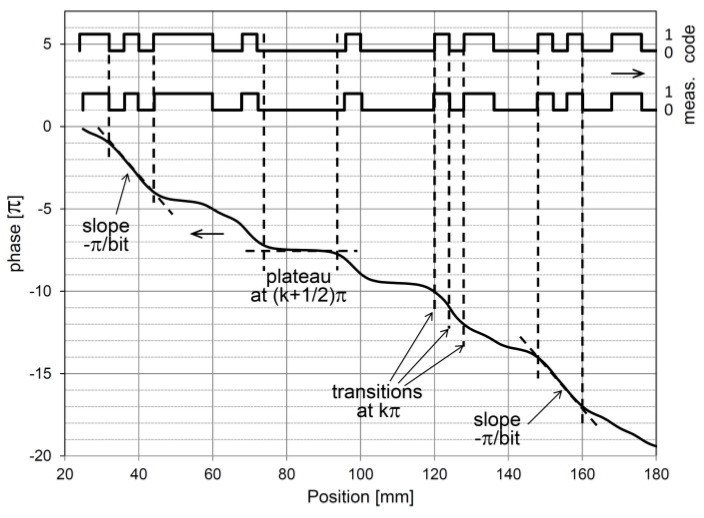
The relationship between the magnetic field vector angle and the position for an exemplary bit pattern (falling curve). The position dependencies of the encoded bit values and the sensed bit values are also illustrated.

The sensing head comprising the magnetic field sensing array measures a set of magnetic flux density vectors, referred as measured field pattern. Unless the magnetic domain fields become indistinct at greater distances above the code tape, a sensing element moving along the tape will experience a consistent rotation of the magnetic field vector in one direction. For instance, as depicted in Figure 2, when moving from left to right above the tape, the measured field vector rotates clockwise. The vectors are nearly vertical near the centers of the domains and almost horizontal near the boundaries where the polarization direction changes. The exact positions where the vectors are vertical or horizontal deviate from the true magnetic polarity boundary positions due to neighboring symmetries. Based on this observation, the 0→1 and 1→0 transitions can be approximately localized, and corrections can be made using the values of the surrounding bits. Figure 3 illustrates the relationship between the vector angle and longitudinal position. The plateaus at (k + 1/2)π (where k is an integer) correspond to uniform–polarity domains where the magnetically represented bit remains constant. In this example, the uniformly magnetized domains representing the code bits had a bit length of 4 mm. The bit transitions are roughly located at the positions where the curve intersects the integer multiples of π. Specifically, for even and odd *k* values, the respective transitions 0→1 and 1→0 can be identified. In the domains where the code bit alternates, steep slopes of -π/bit are observed, which can also be used to autonomously determine the bit length if it is not known from other sources.

Since the magnetic field is sensed by an array of sensing elements, additional uncertainty arises due to the finite spatial resolution of the measured field pattern. Although the bit transition positions are only approximately determined within the coordinate system of the sensing head, this uncertainty remains within a certain proximity to the code tape. Even with linear interpolation of the vector angle values between the sensing elements, the uncertainty does not exceed approximately 30% of the bit length. For instance, with a 1 mm-thick code tape, a 4 mm bit length, and a constant 5.6 mm distance between the sensing elements, this holds true up to 3.5 mm from the code tape.

It should also be noted that, in our approach, when evaluating the bit pattern covered by the sensing array from the measured field pattern, the distance between the sensing elements does not need to have a specific relationship with the magnetic bit length, allowing for the use of different code tapes. Additionally, a constant distance between neighboring sensing elements is not strictly necessary. This flexibility is very useful for miniaturization or when manufacturing inaccuracies occur. However, it is straightforward and common to distribute the sensing elements equidistantly.

The only condition we strictly require is a constant length of the magnetic bits. Any variations are handled by the self-learning algorithms discussed later in this manuscript.

**Figure 4:**
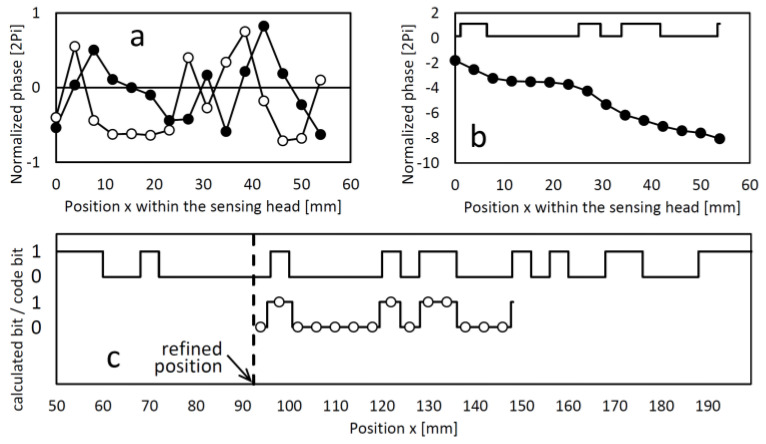
(**a**): The x and z components of the measured field pattern (full and hollow symbols, respectively). (**b**): The calculated vector angles at the relative positions of the sensing elements and the code bit levels derived from the kπ transition points. (**c**): The map of the code bits (top signal) and the sensed bit levels with the identified bit pattern (bottom), shifted to the refined position according to the identified bit pattern within the code bit map and the fractional relative grid position within the sensing head.

To the approximate relative bit change positions in the sensing head’s coordinate system, i.e., the positions defined by crossing integer multiples of π, a grid with a spacing equal to the bit length can be fitted. Not all positions on this grid will correspond to a bit change position. In the middle of the grid intervals, the magnetic bits are determined by discriminating between 0→1 and 1→0 transitions, enabling the identification of the magnetically coded bit pattern covered by the sensing head. Using a known code generator algorithm, such as a 12-bit maximum-length sequence, the coarse position (with one bit-length resolution) can be calculated. Alternatively, if the complete reference bit map of the code tape is stored in the sensor memory, the bit pattern can be searched either within the proximity of the most recent position or globally. Additionally, when fitting the grid, the fractional position (in terms of one bit length) of the starting point of the grid is calculated, providing a refined positioning of the sensing head. This workflow is illustrated in Figure 4.

**Figure 5:**
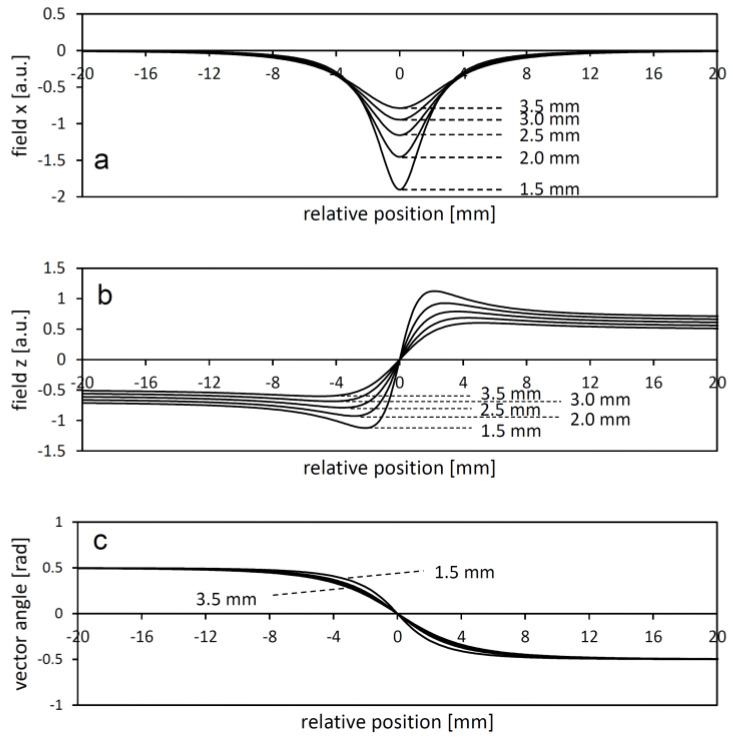
(**a**,**b**): The x and z components of the magnetic field at a certain distance above the magnetic code tape (distance indicated in the plots), shown as a function of the relative position from the boundary between very wide magnetic domains. On both sides of the boundary, the polarization is uniformly vertical and of equal magnitude, differing only in direction. (**c**): The relationship between the corresponding vector angles and the relative position.

Further refinement of the actual bit transition position can be achieved by analyzing the neighborhood of the bit transition positions. Figure 5 illustrates the magnetic field components and the field vector angle near the boundary between two large homogeneous tape domains at different heights z. Note the spatial extension of the field, especially at greater distances. In this example, the magnetic bit length is 4 mm, indicating that multiple bit coding domains may influence the actual magnetic field at a real bit change point.

**Figure 6:**
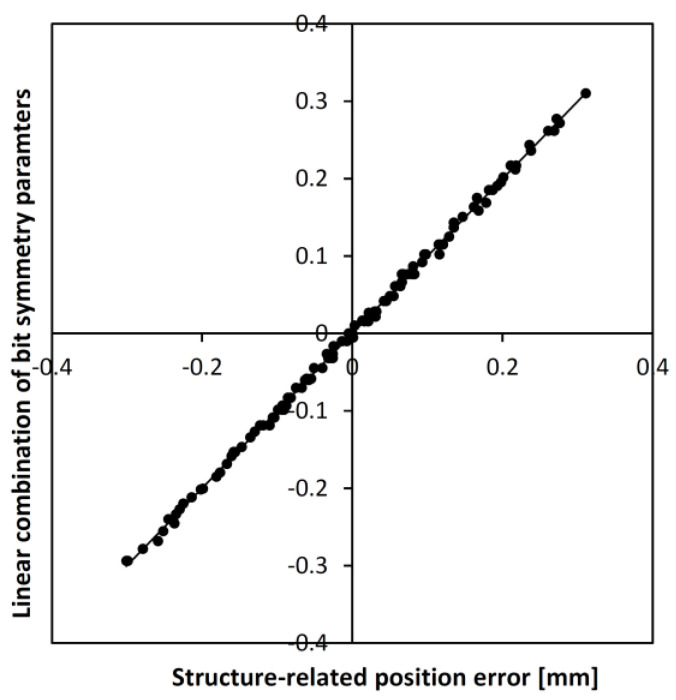
The correlation between the structure-related magnetic bit transition position error and the linear combination of the symmetry parameters for an 8-bit MLS code. The distance from the tape was z = 2 mm.

The influence is minor, so we treat this as a perturbation of the field angle and the associated measured position of the bit transition boundary (defined by crossing integer multiples of π). We search for the structure-related position error as the linear combination of symmetry parameters corresponding to the pairs of bits located left and right of the 0→1 transition point (excluding the first bits, considered to be 0 and 1, respectively). Let b_Lk_ and b_Rk_ be the k-th bit values to the left and right of the boundary, and define the k-th symmetry parameter as a_k_ = 1 − (b_Lk_ + b_Rk_). From a simulation on a tape encoding an 8-bit maximum-length sequence, we found that for any height between 1.5 and 3.5 mm, the structure-related position error E can be expressed as $E=\sum_{k=2}^{6}e_{k}\left(z\right)a_{k}$. The coefficients are shown in Table 1. The parameter (z) refers to the z dependence of the coefficients, so if the expected accuracy in an actual application requires correction of the structure-related position errors, the actual value of z must be known or determined from the measurements themselves. Several methods can be used, ranging from analyzing the field vector magnitudes to examining the z vs. x trajectories. In this case, the bit length was 4 mm, and the thickness and width of the tape were 1 mm and 10 mm, respectively. In a different structure, the coefficients may vary. An exemplary correlation between the structure-related position error and the most appropriate linear combination of the symmetry parameters is shown in Figure 6.

Due to the consistent rotation of the magnetic field vector above the magnetic code tape, the bit pattern can be recognized even if the distance between the sensing elements is greater than the bit length (but still less than two bit lengths). However, because the bit inversion points between two sensing elements can always be estimated through some form of interpolation, a greater distance results in a higher ultimate error.

**Figure 7:**
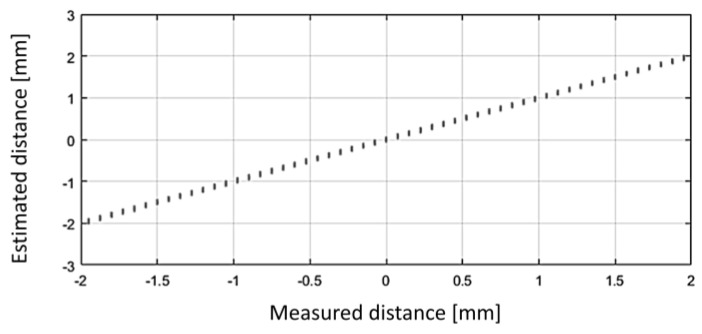
Estimation of sensor displacement through an artificial neural network (ANN) trained on a collection of pairs of measured field patterns.

The local linear error can be minimized further by utilizing an artificial neural network (ANN) to precisely evaluate the dissimilarity between two positions, using two acquired field patterns as inputs for the ANN (Figure 7). In the present example, the array consisted of 32 dual-axis sensing elements spaced at 1.805 mm intervals. The ANN featured a single input layer with 128 neurons and three hidden layers with 20, 15, and 5 sigmoid neurons, respectively. The output layer contained one linear neuron. Within the span of one magnetic bit (approximately 3.93 mm), the output displayed a linear relationship with the pattern distance, and the error followed a normal distribution with a standard deviation of 13 µm. However, the potential effects of reducing the sensor’s spatial resolution and the size of the ANN were not thoroughly investigated.

It should also be mentioned that in our study, we used three-axis sensing elements, but only the x and z components were used in the calculations. The y component was primarily used to detect misalignments and to allow their reduction by rotating the measured field vectors around the *x*-axis. However, even if this rotation is not performed (e.g., when using only two-axis sensing elements), the determination of the measured bit patterns is only slightly affected, as the bit transition positions are associated with the horizontal orientation of the field vectors.

## 3. Adaptation through Learning the Reference Bit Map of the Magnetic Code Tape

A method for converting the measured field pattern into the bit pattern covered by the array of sensing elements was described above. For a matched pair of a sensing head and a magnetic code tape with a known code generator algorithm, the inverse algorithm can be stored in the sensor to decode a measured bit pattern into a coarse position or even the complete sequence of bits represented by the magnetic domains of the code tape, at least within the range of motion. However, it is also possible that the sensor has no prior information about the algorithm to decode the magnetic code tape—meaning it has no algorithm to obtain the coarse position from the measured bit pattern, and a reference bit map is also not provided.

The bit length may be either known or unknown. If autonomous determination of the bit length is required, it is unlikely that the sensor will immediately operate properly in an unknown environment. This is because the grid needed to be fitted to the approximate bit transition boundaries is unknown, and the bits cannot be separated to obtain the measured bit patterns. However, in scenarios such as a maximum-length sequence, a run of alternating bits, at least as long as the slope indicated in Figure 3, statistically occurs at every 32nd position, evenly distributed over the sequence. This segment always represents the part of the vector angle curve with the steepest slope. Once this is found, and the bit length is identified as one of the standard options (e.g., 2, 4, 5, or 10 mm), the sensor can begin learning the reference bit map or operate normally.

**Figure 8:**
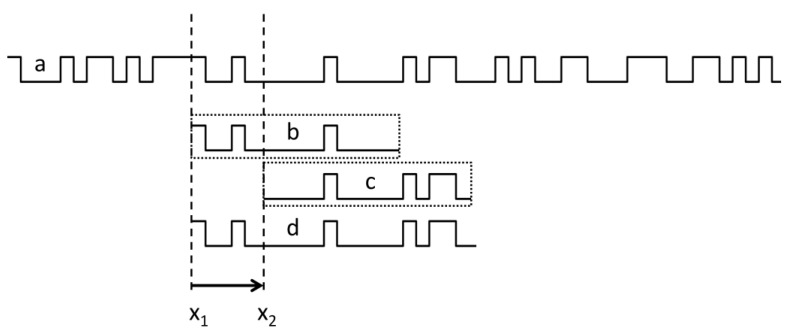
(**a**): An exemplary bit sequence represented by a code tape. (**b**,**c**): The measured bit patterns within the length of the sensor positioned at *x*1 and *x*2, respectively. (**d**): The reference bit map as learned during an initial motion of the sensor from *x*1 to *x*2.

To establish the aforementioned reference bit map through learning, binary values corresponding to the sequence of magnetic bits on the magnetic code tape are stored on the map. Teaching can commence during the initial use of the position sensing system, triggered by user input or when a locally detected bit sequence cannot be matched to a sequence already present in a learned reference bit map. The reference bit map is initially created from a recognized bit sequence and continually expanded during system operation as the sensor head moves within the motion region (Figure 8). The following steps outline the learning process for the reference bit map:

(i) If the reference bit map is empty, the currently detected bit sequence is stored at the beginning of the map;
(ii) The sensor searches for a previously detected and stored bit sequence on the reference bit map;
(iii) If a currently detected bit sequence is found within the reference bit map, the map remains unchanged;
(iv) If the currently acquired bit sequence, truncated at the beginning or end, is not shorter than a matching bit sequence found on the reference bit map, the bits of the truncated portion are appended to the beginning or end of the reference bit map. If new bits are added to the beginning, they are characterized by negative consecutive numbering;
(v) If the truncated bit sequence is not found on the reference bit map, a second reference bit map is created.

In this scenario, another reference bit map is constructed following the steps outlined above. This process continues until a match between the current reference bit map and the second map is found at their respective opposite ends within a sufficiently large area (no shorter than the length of non-repeated sequences). At this point, the two maps are merged into a single map.

**Figure 9:**
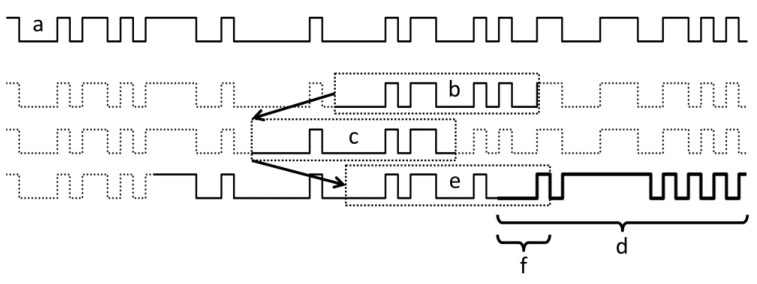
(**a**): An exemplary bit sequence represented by a code tape. (**b**,**c**,**e**): The measured bit patterns within the length of the sensor at different positions. (**d**): The bit sequence represented by the altered portion of the code tape. (**f**): The modified bits in the measured bit pattern.

Figure 9 depicts the identification of a notable alteration in the magnetic code tape as presented in Figure 8. The top section illustrates a previously learned bit sequence stored on a reference bit map (Figure 8a). The lower three lines elaborate on the process of recognizing the change.

While the sensor head remains within an unchanged area of the code tape (Figure 9a), it functions in normal mode, and the measured bit patterns (Figure 9b,c) are easily matched on the reference bit map. If a segment of the code tape is substituted with another segment featuring a differently encoded bit pattern (Figure 9d), bit errors will frequently and systematically occur as the sensor head enters this area. However, if the altered bit patterns (Figure 9e) only consist of a few modified bits (Figure 9f) at the end in the direction of sensor head movement, the bits on the reference bit map corresponding to the altered segment can be marked as unreliable. Thus, the position of the sensor head can still be determined with sufficient accuracy. Consequently, as the sensor head advances further into the altered region, the detected bit pattern will not correspond to any, or only a relatively improbable portion, of the reference bit map.

To enhance the robustness of the sensor setup, it is beneficial to re-enable the aforementioned learning mode even during regular operation or to keep it permanently active. This enables the learning of bit patterns encoded in the modified code tape, potentially as an alternative reference bit map. Consequently, the sensor head position can be determined using both the normal and alternative reference bit maps, selecting the more reliable position value. If the position can be consistently determined with greater accuracy using the alternative reference bit map, it can then replace the normal reference bit map.

In the previous example, the sensor encountered a region where the magnetic state had altered since its last visit, leading to unexpected bits at one end of the measured bit pattern. Nevertheless, the sensor can still ascertain its position by omitting one or two bits and confining the search within the reference bit map to the vicinity of the most recent known position. The “leave out one (or two)” principle can be applied in a broader context for the following scenarios:

(i) If the tape is damaged or remagnetized at a point, resulting in one or two detected bits being uncertain or “blinking,” the corresponding bits in the reference bit map can be labeled as unreliable and excluded from subsequent evaluations;
(ii) If one sensing element is faulty or damaged, providing irrelevant data, the corresponding bits in the measured patterns can be marked as unreliable and omitted from the analysis.

Implementing these measures does not guarantee that the sensor system will maintain the same level of accuracy, particularly if sufficient redundancy is lacking (such as sensing element density or sensor length compared to the nonrepeated code length). However, it is reasonable to expect that the sensor will continue functioning under changed conditions, potentially enabling the preservation of life or value through continued operation. For instance, this could facilitate the removal of materials and workpieces from hard-to-reach areas and the safe shutdown of machinery.

## 4. Determining the Spatial Position of Magnetic Field Patterns

The localization of the sensor relative to the magnetic code tape, as outlined earlier, relies on a reference bit map. This method necessitates uniform lengths of magnetic domains representing the code bits, where both ones and zeros are encoded with consistent magnetic polarizations of equal strengths but opposite directions, perpendicular to the code tape. This level of uniformity allows the magnetic tape to be accurately described solely by the corresponding sequence of bits.

In an alternative localization method, the sensor captures the magnetic field pattern, similar to the previous method. However, instead of computing the measured bit pattern and matching it within a stored or learned bit map, the sensor directly localizes the measured field pattern within a pre-learned magnetic reference field map. This approach allows for less regularity in the code object, should simply be a magnetic item with non-repeating magnetic patterns. However, for demonstration purposes, we will employ a single-track binary absolute magnetic code tape.

**Figure 10:**
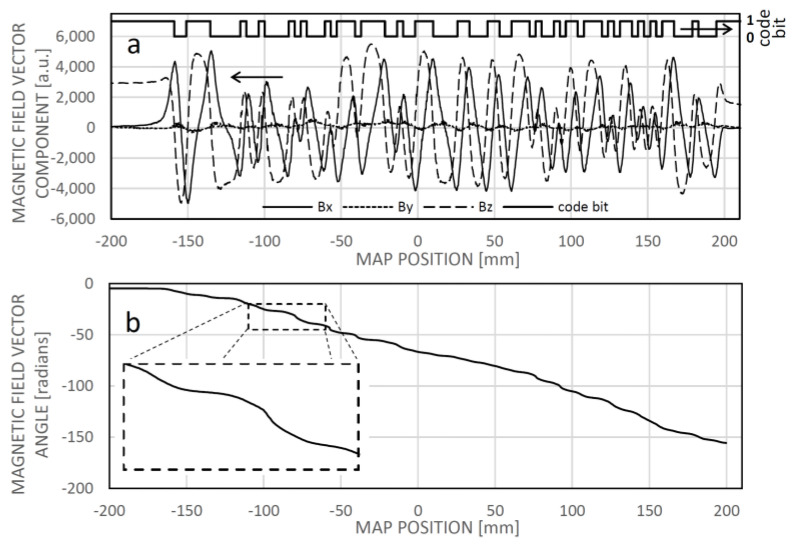
The maps of the magnetic field vector components (**a**) and the vector angle (**b**) captured along a longitudinal trajectory above a magnetic code tape. The corresponding binary sequence is displayed in (**a**).

The magnetic reference field map, or field map, depicts the relationship between the magnetic field vector and the longitudinal position (Figure 10a). Stored within the sensor’s memory, it comprises a series of values corresponding to the x and z components, or potentially the x, y, and z components, of the magnetic field above the tape at a height typical of the sensing elements. Additionally, the vector angle of the magnetic field can also be documented (Figure 10b).

During normal operation, the sensor collects magnetic field vector data from the sensing elements and generates the measured field pattern. Subsequently, it identifies the section of the field map that closely resembles the measured field pattern. Under the assumption that patterns equal to or longer than the sensing array are not repeated within the field map, the position of the most similar section in the map is determined as the actual position of the sensor.

To quantify similarity, an established correlation technique, such as computing the average of squared deviations between the elements of the compared signals using the method of “least squares,” can be employed. Alternatively, other metrics like the average of absolute differences may also be utilized. Normalizing the patterns before assessing dissimilarity is advantageous to mitigate the influence of the distance between the sensor and the code object in the z-direction. A scalar representing the magnitude of deviation between two normalized field patterns can be regarded as a measure of “dissimilarity”, thus we will refer to it as minimum dissimilarity rather than maximum similarity.

**Figure 11:**
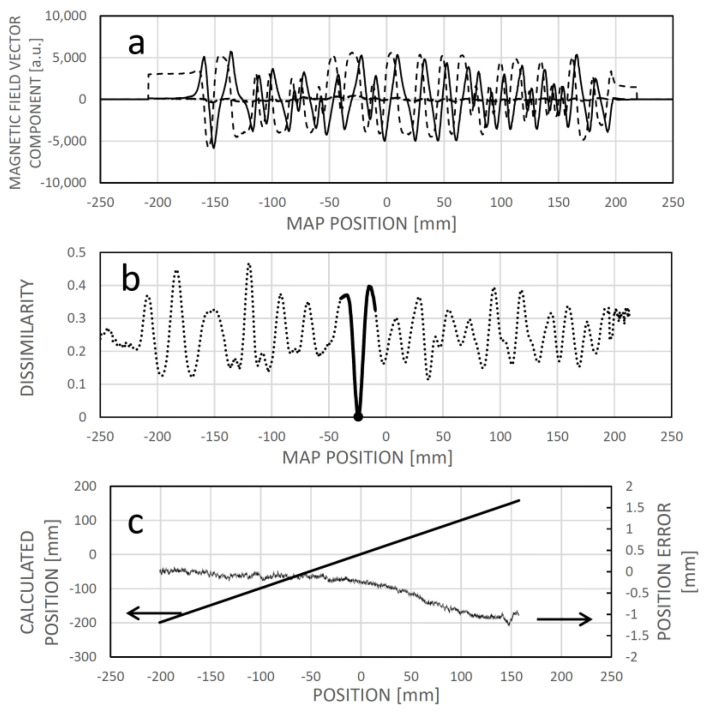
(**a**): Learned field map. (**b**): The dissimilarity between a measured field pattern and field patterns extracted from the field map, depicted as a function of their respective positions in the map. The map location with the minimum dissimilarity is deemed the calculated position. The thick solid section denotes the vicinity of the actual sensor position. (**c**): Correlation between the calculated and the actual (experimental) positions, along with the associated error (the difference between the two). In this instance, the dissimilarity figure was computed as the averaged square difference between the respective elements of the measured field pattern and those extracted from the reference field map for the given map positions.

Assuming the reference field map (Figure 11a) is already established (through learning) for the motion range, an instance of localizing the sensor around −24.2 mm involves identifying the minimum dissimilarity between the sensed field pattern and the corresponding one from the field map at that position (Figure 11b). Figure 11c illustrates the linear nature of the dissimilarity-based calculated position, along with the error accumulated during the learning phase. 

**Figure 12:**
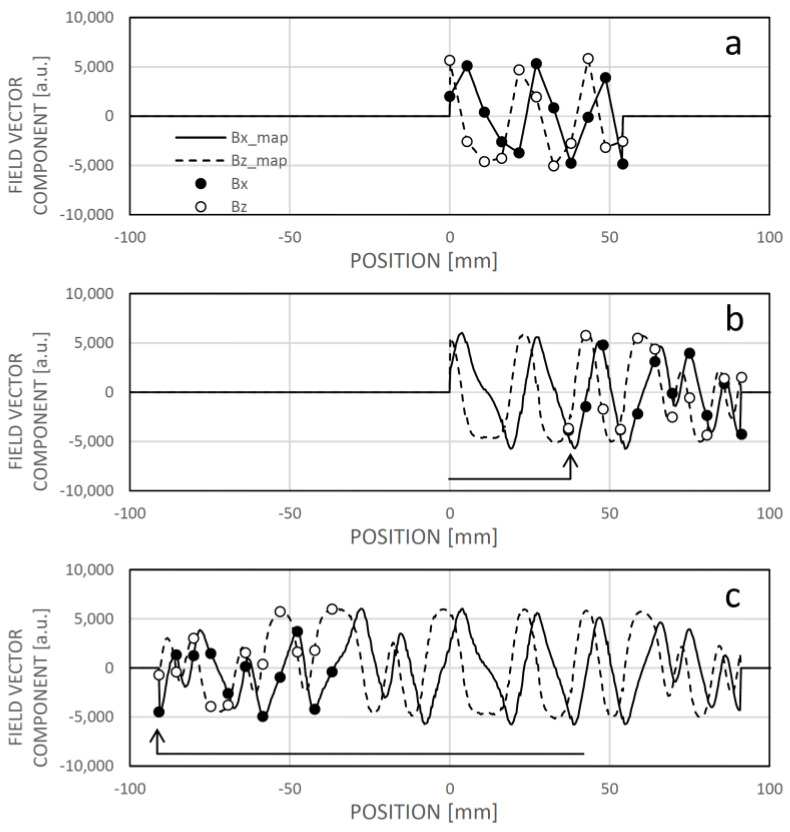
The process of learning the field map. In (**a**), the initial map values are set to zero. The sensor captures the first field pattern and records it into the field map, employing linear interpolation between sensing elements. By default, the actual position may correspond to the zero position in the map. In (**b**), the sensor moves to the right, continually localizing its position within the previously learned map section. The measured field pattern values are integrated into the map based on the calculated position, with intermediate values determined through linear interpolation. Subsequently, (**c**) demonstrates further expansion of the map into the negative direction.

The learning process is depicted in Figure 12, where the initial map values are set to zero. The sensor acquires the first field pattern and integrates it into the field map using linear interpolation between the sensing elements (Figure 12a). By default, the actual position may align with the zero position in the map. As the sensor moves to the right, it continually localizes its position within the already learned map section (Figure 12b). During this movement, the rightmost sensing elements encounter a zone where the reference field map contains constant zeroes. In evaluating the minimum dissimilarity, data from sensing elements continuously compared with constant zeroes does not contribute to localization. However, as the actual position is determined based on partial patterns moving above the known field map part, the “white spots” in the field map are overwritten with the measured field data. Figure 12c illustrates the extension of the field map data on the left side during reverse motion. The sensor system is already operational during learning, even when the reference field map is not yet known.

**Figure 13:**
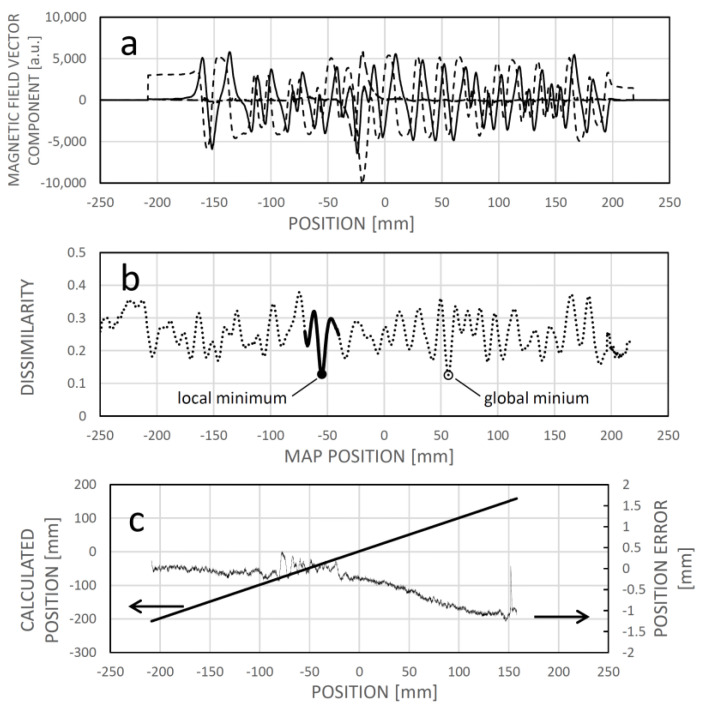
The impact of an interfering magnet situated near the magnetic code tape at approximately −25 mm longitudinal position. In panel (**a**), the actual dependency of the magnetic field vector components on position is shown, with distortion evident between approximately −50 and 0 mm map positions. Panel (**b**) displays the dissimilarity between a measured field pattern and patterns extracted from the field map across their respective positions. Due to field curve distortion, the global minimum of the dissimilarity function does not align with the real sensor location, resulting in only a local minimum. However, given the expectation that the real position is near the previous sensor location, the local minimum can be identified and tracked during motion. Panel (**c**) depicts the correlation between calculated and real sensor positions, revealing increased error between approximately −80 and −20 mm. This domain is not symmetrical to the magnet position (−25 mm) because the sensing array is positioned to the right of the leftmost sensing element, associated with the actual position.

**Figure 14:**
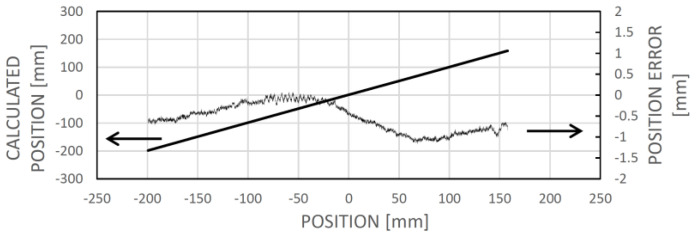
The correlation between the calculated and actual sensor positions, along with the error of position determination, subsequent to relearning the magnetic field map in the presence of the interfering magnet.

In the initial stage of field map creation, the first measured field pattern is recorded into the field map using linear interpolation between the measured points (Figure 12a). As the system begins to move, this interpolated yet imprecise section of the field map is used for localization, causing measured field patterns used for expanding and updating the map to be localized with an error in this early phase. This results in a persistent shift of the longitudinal scale. However, this error can be corrected later by saving the initial field pattern, presumed to correspond to the zero position of the field map, and recalculating its position once the field map is complete. Without this post-correction, zero offsets, as seen in Figure 11c, Figure 13c and Figure 14, may occur. An accumulated integral error can also be observed, particularly in the positive part of the sensing range. This error may stem from various factors, such as inaccuracies in the positions and alignments of the sensing chips, variations in chip sensitivities within the sensing array, noise, and the timing scheme for sampling the magnetic field components to capture the measured field pattern. The impact of these factors was not analyzed in the current study.

In the example depicted in Figure 13a permanent magnet was positioned near the magnetic code tape at approximately −25 mm. Figure 13a illustrates the field vector components as a function of position (which can be regarded as an alternative field map within this context). In Figure 13b, an example of dissimilarity is shown for a sensor position around −54.8 mm, revealing a local minimum rather than a global one. Consequently, when searching for the sensor position based on minimum dissimilarity, it is practical to localize the new position near the last known one where the sensor is likely to be found, by identifying the local minimum of dissimilarity. This approach introduces a minor error while ensuring the sensing system’s continued operation (Figure 13c).

Continuing to operate the learning mode even after accumulating the field map data over the entire range of motion proves practical for two primary reasons. Firstly, it allows for improving the field map by gathering additional measurement data at points initially unsampled during the initial learning phase. Secondly, ongoing learning may yield an alternative reference field map, distinct from the one established through self-learning upon installation. Such discrepancies often signal changes in the magnetic code tape, alterations in the magnetic or ferromagnetic environment, or misalignments of the sensor concerning its original trajectory along the code tape, thereby offering diagnostic insights.

If the observed difference persists between the alternative field map and the operative reference field map, then replacing the operative map with the alternative one allows the sensor to continue operating with a reference map aligned to the changed environment, thereby reducing local errors (as depicted in Figure 14).

**Figure 15:**
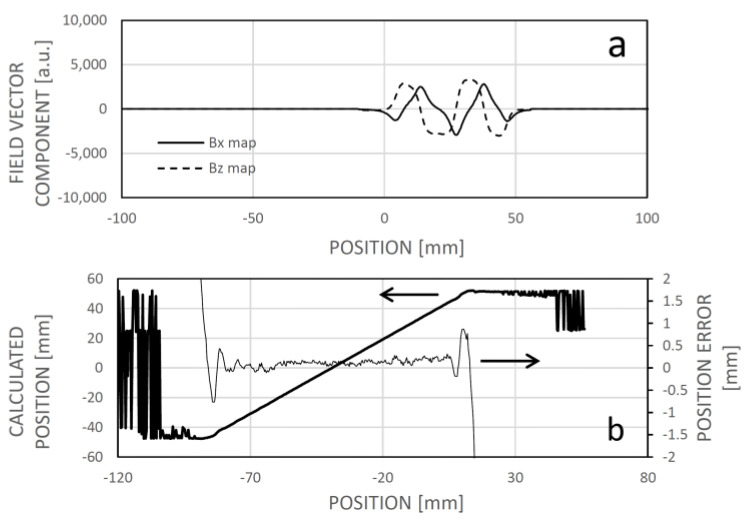
(**a**): The magnetic field map of a brief irregular code object. (**b**): the calculated position attained through a global search for the minimum dissimilarity, alongside the positional error (offset excluded).

The effectiveness of the field map-based positioning technique is further demonstrated in Figure 15. A short and irregularly magnetized magnetic code object was used, consisting of a 4 mm wide and 1 mm thick magnetoelastic stripe. This code was manually created using a neodymium permanent magnet in two steps: first, the stripe was erased by sweeping one pole of the magnet across it, then it was touched at various points by the other pole. The sensing array comprised 11 members with a center-to-center distance of approximately 5.4 mm between adjacent elements. The sensor learned the map during a total movement of 5.6 mm and exhibited linear characteristics over nearly 100 mm. Alignment was done manually to appear parallel with the code stripe. During the recording of characteristics, a global search was forced, treating each point as an evaluated position as if the sensor had just appeared there. 

Concerning the global (integral) error resulting from the cumulative localization error of partial measured field patterns at the boundaries of the learned map parts, one may consider utilizing a regularly structured magnetic code tape containing a pseudorandom binary sequence. It is important to highlight that the field map method demonstration utilized such a code tape, albeit without leveraging its regularity (Figure 11c, Figure 13c and Figure 14). Operating in parallel, the bit map, with its structured longitudinal grid, can aid in error cancellation and offer supplementary position information during initial learning. Meanwhile, field map-based localization can navigate through damaged or altered domains. Additionally, integrating ANN-based displacement sensing can enhance local linear properties.

For the autonomous learning of the reference bit map and determination of the sensor position, a Cortex M4 based microcontroller operating at 80 MHz with 64 kB RAM and 32 kB FLASH was effectively utilized. While the field map-based localization method requires significantly more memory to store the reference map—approximately 40 bytes per magnetic code bit for 10 times spatial resolution and 12- to 16-bit magnetic field component data—it also demands greater computational power. This is achievable only in Cortex H7 or similar devices, or in FPGAs, where operations can be effectively parallelized. In our study, the field map-based method was operated in a “hybrid” manner, using the sensor head solely for signal acquisition.

**Figure 16:**
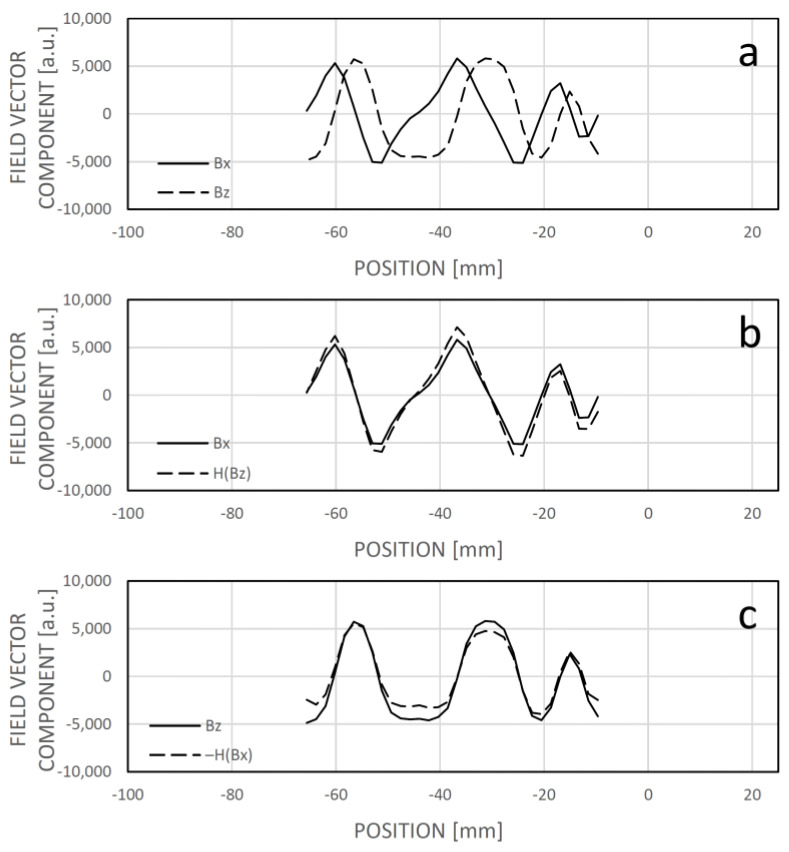
(**a**): The measured field pattern at a random point along the trajectory. (**b**): The x component and the Hilbert transform of the z component of the field pattern. (**c**): The z component and the negative Hilbert transform of the x component of the field pattern.

The bit map technique described previously allows for the calculation of the original code bit sequence from the field maps, as shown in Figure 10. This capability facilitates a seamless transition between the bit map and field map techniques, enabling cross-diagnostic functions between the two methods. Additionally, the spatial dependencies of the x and z components exhibit nearly harmonic conjugates, which can be aligned using a linear integral transformation, such as the Hilbert transformation. This alignment improves their phase-related match or overlap, allowing for joint evaluation in determining a corresponding bit pattern, even if the sensor elements are sensitive to only one field vector component. For instance, when elements are sensitive solely to the x component, the z component can be approximated by applying a Hilbert transformation to the Bx component (Figure 16). On small samples, such as the measured field patterns, the Hilbert transform can be efficiently computed via convolution and can run in real time on both microcontrollers and FPGA architectures. It is important to note that the field map-based position determination method also works effectively with single-axis sensing chips without generating the harmonic conjugate of the field pattern. This approach may help reduce manufacturing costs as well as data acquisition and processing time, although it requires further analysis. For practical reasons, maintaining the sensitivity of the magnetic field sensing chips in the x direction is advisable, as this vector component is the least sensitive to lateral displacement, yaw, and rotation of the sensor.

## 5. Conclusions

In this paper, we presented a self-adaptive approach to absolute position sensing using a single-track magnetic code tape. We introduced two methods for learning reference maps of the code tape: one assuming the tape represents a regular sequence of magnetic bits (reference bit map method) and another for a more general code object (reference field map method). The combination of these methods, along with an artificial neural network-assisted short-range displacement estimation, provides a robust, error-tolerant, and adaptive solution for long-range linear position sensing. This approach can be used independently or as a reliable backup system. Further investigations will focus on improving the system’s linearity and resolution.

## Figures and Tables

**Table 1:** The coefficients of the polynomial describing the structure-related position error.

z [mm]	e2 [mm]	e3 [mm]	e4 [mm]	e5 [mm]	e6 [mm]
3.5	−0.060	−0.141	−0.075	−0.042	−0.026
3.0	−0.137	−0.121	−0.059	−0.032	−0.020
2.0	−0.185	−0.077	−0.032	−0.016	−0.010
1.5	−0.162	−0.054	−0.021	−0.011	−0.006

## Data Availability

The dataset is available on request from the authors.
